# Automaticity in Stimulus-Parity Synaesthesia

**DOI:** 10.1177/2041669517736323

**Published:** 2017-11-02

**Authors:** Tsvetomira Dumbalska, Rebekah C. White, Mihaela D. Duta, Kate Nation

**Affiliations:** Department of Experimental Psychology, University of Oxford, Oxford, UK

**Keywords:** categorisation, colour, shape, synaesthesia

## Abstract

Automaticity is a defining characteristic of synaesthesia. Here, we assess for automaticity in stimulus-parity synaesthesia; a subtype that has been documented only 3 times in the literature. Synaesthete R experiences many (nonnumerical) stimuli as being odd or even. She described a toy shape-sorter, which paired odd shapes with even colour slots (and vice versa) and relayed difficulties with the incongruency created by this simple toy. Inspired by this anecdote, we devised a computerised task in which Synaesthete R (and 10 control participants) indicated the location of a target shape, which was presented on a coloured bar. Synaesthete R (but not control participants) was faster to report the location of target shapes presented on colours of congruent synaesthetic parity, relative to target shapes presented on colours of incongruent synaesthetic parity. These results constitute the first objective demonstration as to the automatic nature of associations in stimulus-parity synaesthesia.

## Introduction

Individuals with synaesthesia experience atypical merging of sensory and cognitive constructs (Simner, 2012). The stimulus that gives rise to the synaesthetic experience is referred to as the *inducer* and the synaesthetic experience itself is referred to as the *concurrent*. Example subtypes of synaesthesia include sound-colour synaesthesia, in which music and other sounds elicit colour sensations (e.g., Ward, Huckstep, & Tsakanikos, 2006); lexical-gustatory synaesthesia, in which words elicit flavour and food sensations (Ward & Simner, 2003); and grapheme-colour synaesthesia, in which graphemes elicit colour sensations. Not surprisingly, synaesthetes are described as having an “enriched world of experiences” (Meier & Rothen, 2013, p. 76).

Synaesthetic associations can be pleasant; for example, sound-colour synaesthetes report that colours enhance their musical experience, allowing them “to enjoy music even more” (Glasser, 2015, p. 5). And so too can synaesthetic associations be helpful. Synaesthetes make use of their concurrents when learning about new stimuli (see Watson, Akins, Spiker, Crawford, & Enns, 2014) and may report enhanced memory for stimuli which induce synaesthetic experiences (Rothen, Meier, & Ward, 2012), perhaps because the concurrents are used as mnemonic devices (Watson et al., 2014). Indeed, a synaesthetic advantage in performance on various memory tasks has been demonstrated for colours (e.g., Yaro & Ward, 2007), words (e.g., Radvansky, Gibson, & McNerney, 2011), abstract figures (e.g., Rothen & Meier, 2010) and events (e.g., Simner, Mayo, & Spiller, 2009).

Sometimes, however, synaesthetic associations can make daily activities unpleasant; for example, while reading or engaging with conversations, lexical-gustatory synaesthetes may experience strong, persistent and long-lasting tastes (Ward & Simner, 2003). And, so too can synaesthetic associations make daily activities difficult. One synaesthete, who experienced colours for multiple stimuli, was unable to play the piano because she experienced incongruencies between three sets of synaesthetic colours – first, a set for her fingers; second, a set for musical pitch; and third, a set for the letter name associated with each note. She turned to learning an alternative instrument and used strategies to overcome the interference caused by her synaesthesia (Pautzke, 2010, cited in Watson et al., 2014)

These latter examples highlight the automatic and involuntary nature of synaesthetic associations. Indeed, this is one of the defining characteristics of synaesthesia. In the laboratory, the automaticity of synaesthetic associations has been assessed using various tasks. These include adaptations of the Stroop task (Stroop, 1935) as well as reaction time priming tasks. In the Stroop task, an automatic and rapid process (i.e., reading) comes into conflict with another more controlled and effortful process (i.e., naming ink colours). In the original Stroop task, participants were presented with colour words, and the task was to report the ink colour in which the colour words were printed. The colour word and ink colour could be congruent (e.g., ‘green’ written in green ink) or incongruent (e.g., ‘green’ written in yellow ink). When the colour word and the ink colour were congruent, participants were fast to name the ink colour. In contrast, when the colour word and ink colour were incongruent, participants were slower to name the ink colour. The interpretation of this robust finding is that participants automatically read the colour word as it is presented and are slowed by the conflict created by an incongruent ink colour.

More than 30 years have passed since Wollen and Ruggiero (1983) made the first strides in adapting the *Stroop task* for testing the automaticity of grapheme-colour synaesthetic associations. Letters were presented in an ink colour that was congruent or incongruent with the synaesthetic colour, and the participant’s task was to report the ink colour. Wollen and Ruggerio, as well as numerous researchers since (e.g., Lupiánez & Callejas, 2006; Mattingley, Rich, Yelland, & Bradshaw, 2001), demonstrated a robust Stroop effect. Synaesthetes were faster to name the ink colour when the synaesthetic letter colour and the ink colour were congruent as compared with incongruent (for a review of grapheme-colour synaesthesia, see Hubbard & Ramachandran, 2005). Variations on the Stroop task have been used to investigate numerous subtypes of synaesthesia, and Stroop effects have been demonstrated for digit-colour synaesthesia (Mills, 1999), sound-colour synaesthesia (Ward et al., 2006), sound-taste synaesthesia (Beeli, Esslen, & Jäncke, 2005), mirror-touch synaesthesia (Banissy, Cohen Kadosh, Maus, Walsh, & Ward, 2009), ordinal linguistic personification (Simner & Holenstein, 2007) and swimming-style colour synaesthesia (Nikolić, Jürgens, Rothen, Meier, & Mroczko, 2011).

Rothen, Nikolić, Jürgens, Mroczko-Wąsowicz, Cock, Meier (2013) have also used a *reaction time priming task* to investigate automaticity effects in swimming-style colour synaesthesia. In this subtype, swimming styles are associated with vivid colour sensations (Nikolić et al., 2011). To assess for automaticity, a picture of a swimming style was presented as a prime, then a colour patch was presented as the target, and the task was to indicate the colour. The synaesthete was faster to indicate the target colour when the prime (i.e., swimming style) was congruent as compared with incongruent. This was also the case when the colour was presented as the prime and the swimming style was presented as the target, with the task of indicating the swimming style.

**Figure 1.**
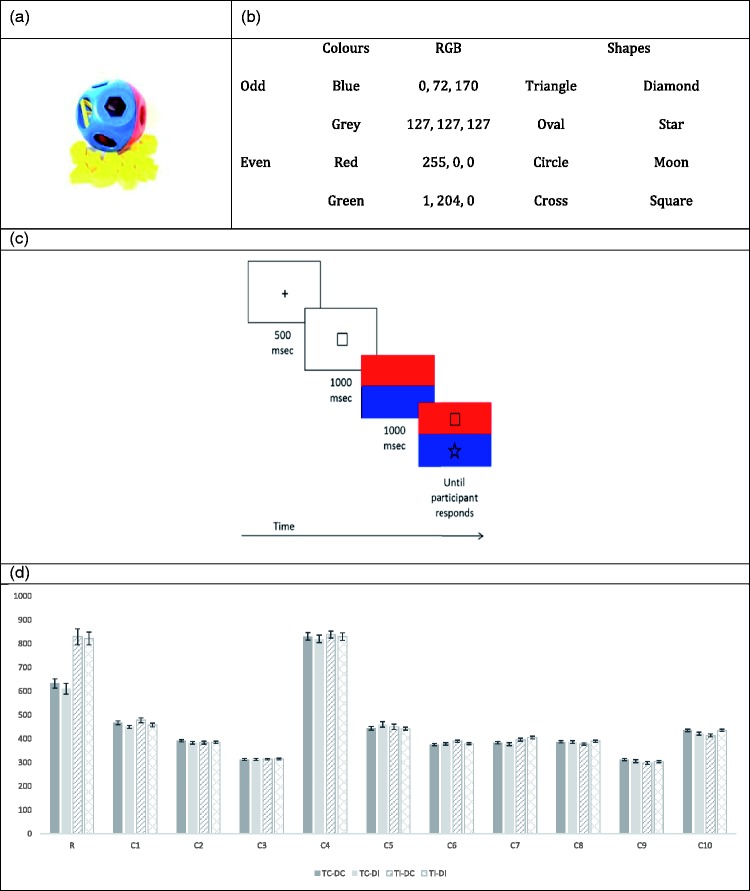
(a) The shape sorter that provided the inspiration for this experimental investigation. (b) The stimuli (shapes and colours) used for the computerised task. (c) An example target congruent-distractor congruent trial, with the even (target) square on the even colour red and the odd (distractor) star on the odd colour blue. (d) Each participant’s mean reaction times (ms) – Synaesthete R and the 10 control participants (C1–C10) – for each of the four trial types: TC-DC, TC-DI, TI-DC and TI-DI. Error bars represent 1 SEM.

Here, we follow Rothen et al. (2013) in using a reaction time priming task to test for automaticity effects in stimulus-parity synaesthesia, a subtype that has been documented only 3 times in the literature (Flournoy, 1893; White, Dumbalska, Duta, & Nation, in press; White & Plassart, 2015). The design of our paradigm is inspired by an anecdote that a stimulus-parity synaesthete shared during a testing session. Synaesthete R reported that her stimulus-parity associations can interfere with her day-to-day activities and she provided the example of a difficulty created by a toy shape sorter belonging to her children. The shape sorter was spherical, half blue and half red (see Figure 1(a)). For Synaesthete R, blue is a strongly odd colour and red is a strongly even colour. Her difficulties emerged because the parity of some shapes was incongruent with their colour slot; for example, the star which is an odd shape had its slot on the red (even) side of the sphere. When putting shapes in the toy, Synaesthete R reported being much slower finding the slots for the incongruent shapes. Thus, we set out to test her subjective impression of inducing stimuli eliciting automatic and involuntary parity associations by devising a computerised colour-shape matching task. Automaticity is a defining characteristic of synaesthesia, and given that research on stimulus-parity synaesthesia is in its infancy, the demonstration that parity associations occur automatically is important to establishing the genuineness of this subtype.

## Methods

### Participants

Synaesthete R is a 33-year-old right-handed woman whose native language is English. She is highly educated, holding a PhD in Physics. She has various subtypes of synaesthesia (a) sequence-space synaesthesia, with letters, numbers, time, weekdays and months occupying three-dimensional shapes in the mind’s eye and around the body; (b) grapheme-colour and lexical-colour synaesthesia, with a small selection of letters, numbers, weekdays and months having colours and (c) stimulus-parity synaesthesia, with letters, numbers, weekdays, months, colours, shapes and words eliciting feelings of oddness and evenness. Consistent with the familial nature of the phenomenon, Synaesthete R’s father is also a synaesthete. As part of a different investigation looking at whether parity is tied to perceptual or conceptual features of a stimulus (White et al., in press), we assessed the consistency of Synaesthete R’s parity associations over time. Most relevant to the current study are her parity associations for shape outlines and colour stimuli. When given a surprise retest at 6 months, Synaesthete R provided consistent parity associations for 8/8 shape outlines (100%), and when given a surprise retest at 11 months, Synaesthete R provided consistent parity associations for 95/100 colour stimuli (95%).

Synaesthete R reports that her parity associations can assist her with remembering information. For example, when attempting to recall a person’s name or a phone number, she might remember there being an associated feeling of oddness, and this cue allows her to narrow the possible options (for a discussion of the helpfulness of synaesthetic associations, see Watson, Chromy, Crawford, Eagleman, Enns, & Akins, 2017). For Synaesthete R, odd stimuli give a dark feeling, whereas even stimuli give a warm and light feeling. But, two odd stimuli will not necessarily elicit the same feeling and nor will two even stimuli. The term oddness encapsulates a range of feelings, as does the term evenness.

In addition to Synaesthete R, we also tested 10 control participants (six women and four men, 18–36 years) who did not have synaesthesia. Control participants were recruited from the University of Oxford community and received £7 compensation for their time. The rationale behind testing control participants was to ensure that any automaticity effects demonstrated by Synaesthete R were due to her synaesthesia, rather than lining up with how most people might perform on this reaction time task.

### Stimuli

The stimuli – inspired by the shape-sorting game – were odd and even coloured bars and odd and even shapes (see Figure 1(b)). The shape images varied from 3.37° to 5.93° visual angle in width and 5.58° to 5.79° visual angle in height. We used two colour pairs – blue–red (in block 1) and green–grey (in block 2) – to rule out the possibility of any effects being driven by direct links between shape and colour, rather than by parity. Stimuli were presented on a 23-inch monitor using custom MATLAB code with Psychophysics Toolbox (Brainard, 1997) extensions, and responses were collected using a Cedrus RB 540 response box.

### Procedure

An example experimental trial is presented in Figure 1(c). Trials began with a fixation point (500 ms), then a target shape was presented (1000 ms) followed by two horizontally oriented coloured bars (1000 ms). Subsequently, two shapes appeared – one on the top bar and one on the bottom bar. These shapes remained on the screen until the participant made a response – using the top and bottom buttons of the response box – to indicate which of the two shapes (top or bottom) matched the target shape. Participants were asked to respond as quickly and as accurately as possible.

There were 224 trials in each block. Half were *target congruent (TC) trials*, with the target shape on a congruent colour and the other half were *target incongruent (TI) trials*, with the target shape on an incongruent colour. Note that our task also involved a distractor shape – the shape which also appeared on the second coloured bar. The distractor shape could itself be on a congruent colour or on an incongruent colour. Thus, there were four trial types: (a) target-congruent and distractor-congruent, (b) target-congruent and distractor-incongruent, (c) target-incongruent and distractor-congruent and (d) target-incongruent and distractor-incongruent. The composition of experimental trials was fully counterbalanced. We predicted that Synaesthete R would be faster to respond to TC than TI trials. We did not have specific predictions for distractor congruency, but we were open to the possibility that an incongruent distractor would make the display generally less pleasant, thereby slowing reaction times. We predicted that control participants would not show any congruency effects, as the notion of parity congruent shapes and colours should not apply to individuals without stimulus-parity synaesthesia.

## Results

### Data Screening

Incorrect responses – 2 trials for Synaesthete R and 86 trials for control participants – were excluded from the data set. Subsequently, the data were screened for outliers, defined as data points lying 3 SDs below or above the given participant’s mean reaction time. This approach resulted in additional trials – 3 trials for Synaesthete R and 60 trials for control participants – being excluded from the data set. As reaction times were not normally distributed, we carried out logarithmic transformations of the data prior to analysis.

We observed that, on average, Synaesthete R’s reaction times (*M* = 741.6 ms, *SD* = 396.2 ms) were appreciably slower than the mean reaction times of control participants (*M* = 440.7 ms, *SD* = 135.7 ms); although note that her mean reaction times were nonetheless within the range (*min M* = 312.9 ms, *SD* = 43.5 ms; *max M* = 840.8 ms, *SD* = 257.2 ms) of control participants.^1^ This general pattern – slower overall reaction times for the synaesthete compared to control participants – has been observed in previous studies using Stroop-like tasks to assess the automaticity of synaesthestic associations (e.g., grapheme-colour synaesthesia: Lupiánez & Callejas, 2006, see their Figure 1; mirror-touch synaesthesia: Banissy et al., 2009, see their Figure 2). In the case of the current investigation, Synaesthete R’s slower reaction times may be partly explained by her age. Reaction times are known to slow with age (e.g., Langan, Peltier, Bo, Fling, Welsh, & Seidler, 2010; Woods, Wyma, Yund, Herron, & Reed, 2015), and Synaesthete R was older than 9 of 10 control participants. In addition, Synaesthete R was cautious in her responses at the outset of the experiment, and became faster as she progressed through the trials; that is, she demonstrated a strong negative correlation between trial number and response time, *r* = −.646, *p* < .01.

### Data Analysis

We conducted separate 2 × 2 analysis of variances on each participant’s reaction times with target (congruent and incongruent) as the first factor and distractor (congruent and incongruent) as the second factor. We did not group control participants, as we wanted to be sensitive to detecting any single participant demonstrating effects for target congruency. We followed Rothen et al. (2013) and treated different trials of one condition as different subjects. Rothen et al. acknowledge that this design violates the assumption of independence of data points; however, it is more conservative than a repeated-measures approach. Results were analysed against a conservative α level of .0045 (.05/11) to adjust for our use of multiple comparisons. Note that for the analyses reported here, we combined the data from the two experimental blocks (blue–red and grey–green). However, the pattern of results was the same when we analysed each block separately, and indeed, when we analysed each colour separately.

#### Synaesthete R

In line with our main prediction, there was a significant main effect for target, *F*(1, 439) = 68.304, *p* < .001, partial η^2 ^= .14, which is explained by Synaesthete R being faster on congruent (*M* = 623 ms, *SE* = 15.6 ms) compared with incongruent (*M* = 823.8 ms, *SE* = 21.4 ms) target trials (Figure 1(d)). There was no main effect for distractor, *F*(1, 439) = .549, *p* = .459, partial η^2 ^= .001, and nor was there an interaction between target and distractor, *F*(1, 439) = .280,* p* = .597, partial η^2 ^= .001.

#### Control participants

The comparison between Synaesthete R and control participants is important in demonstrating that Synaesthete R’s pattern of responding is due to her synaesthesia and does not line up with how individuals without synaesthesia respond. Using Synaesthete R’s system for coding congruency, we found that no single control participant demonstrated a main effect for target (all *p* values ≥ .102) or distractor (all *p* values ≥ .047), nor an interaction between target and distractor (all *p* values ≥ .037).^2^ Response profiles for Synaesthete R and each of the 10 control participants are depicted in Figure 1(d); the reader will appreciate that, whereas Synaesthete R’s mean reaction times across the four trial types varied considerably (218 ms), control participants showed very little variation (range 2 ms [C3] to 28 ms [C1]).

## Discussion

Synaesthete R’s impression of slower processing of parity-incongruent stimuli in her day-to-day activities is beautifully complemented by the data from our reaction time task. She is affected by the parity of colours and shapes, even when this dichotomisation is *irrelevant* to the spatial shape-matching task. The results of the present experiment showed that Synaesthete R was slower to indicate the location of the target shape (top or bottom) when the target shape’s synaesthetic parity (odd or even) was incongruent with synaesthetic parity of its background colour. It is important to note that this was a straightforward and objective task (“is the target shape at the top or the bottom?”), calling for speeded response times. The findings suggest that the parity of the target shape and the parity of the background colour are automatically and involuntarily generated, even when this makes the task more difficult. One might question whether our effects for target congruency are the result of a task-induced strategy, or *involuntary* shape-parity and colour-parity associations? We think a strategic explanation is unlikely. When presented with a shape followed by two coloured bars, there was no benefit to be gained by placing attention on the parity-congruent colour. Indeed, doing so would be to Synaesthete R’s detriment, due to the equally frequent presentation of congruent and incongruent trials. That Synaesthete R was unable to ignore the irrelevant colour information sits more squarely with an explanation based on automatic associations.

In addition to testing Synaesthete R, we also assessed 10 control participants without synaesthesia. As predicted, none of the control participants showed an effect for target congruency. Thus, Synaesthete R’s data do not line up with how individuals without synaesthesia respond. Orne (1962) introduced the term *demand characteristics* to refer to cues that a participant uses to deduce the objectives of an experiment. Orne observed that participants who were able to verbalise the hypothesis of one of his experiments produced the expected experimental effect, whereas participants who were unable to verbalise the hypothesis did not produce the expected experimental effect (Orne, 1959). He surmised that, as far as participants are able, they will behave as “good subject[s]” … “to validate the experimental hypothesis” (Orne, 1962, p. 778). Bearing this in mind, we acknowledge that our task was inspired by Synaesthete R’s self-reported difficulty with a shape-sorter toy, and she knew that she was recruited to our experiment due to her synaesthesia. It is, therefore, possible that she had insight as to our hypotheses (and certainly more insight than control participants) and was motivated to produce a particular distribution of reaction times. However, we also note that the experiment was conducted more than an year after she mentioned her difficulty with the shape-sorter toy, and we did not refer to the shape-sorter toy in our invitation to participate, nor our task instructions. Moreover, the experiment mapped onto the shape-sorter toy in a fairly abstract way: it involved two additional colours (green and grey) and a different assortment of shapes. At the end of the experimental session, Synaesthete R confessed that she was not sure what we assessing, and that although some trials were more jarring than others, she did not believe that this had affected her speeded responses. Synaesthete R’s reaction time difference for TC versus TI trials (*M* = 218 ms) sits comfortably with those from studies investigating automaticity effects in other subtypes of synaesthesia (e.g., Banissy et al., 2009; Lupiánez & Callejas, 2006; Rothen et al., 2013), leading us to favour an interpretation based on interference caused by the involuntary elicitation of synaesthetic concurrents, rather than a more voluntary process.

In previous studies, we have shown that Synaesthete R is highly consistent in her stimulus-parity associations over time (White & Plassart, 2015; White et al., in press). Here, we add another important piece of evidence as to the genuineness of the subtype, by demonstrating the automaticity of Synaesthete R’s stimulus-parity associations. The implications of our work extend beyond this demonstration. Our work contributes an experimental method to assess congruency effects for concurrents that are conceptual in nature. Instead of presenting an inducer in a format that is congruent or incongruent with its synaesthetic concurrent, we paired two inducers (i.e., shape and colour) which had either the same concurrent (e.g., oddness) or two different concurrents (i.e., one was odd and one was even). This approach could be extended to the assessment of other subtypes of synaesthesia. For example, some synaesthetes personify inanimate objects, such as letters, numbers, simple shapes and furniture (see Carriere, Malcolmson, Eller, Kwan, Reynolds, & Smilek, 2007; Smilek, Malcolmson, Carriere, Eller, Kwan, & Reynolds, 2007). Inducing stimuli may be attributed with gender as well as personality types. Simner and Holenstein (2007) used a variation on the Stroop task to assess whether letters are automatically attributed with gender, by presenting names that were either congruent or incongruent with the synaesthetic gender of the first letter (e.g., Brian would be a congruent stimulus for a Synaesthete who reports that B is male). But, assessing whether stimuli are automatically attributed with *personality types* is less straightforward, as personality is conceptual and does not always have an obvious physical representation. We suggest that an adaptation of our spatial paradigm may overcome this difficulty and that researchers may test for automaticity effects using a spatial decision-making task in which the synaesthetic personality of paired stimuli at the target location is either congruent or incongruent.

## Notes

1 Note that there was a speed-accuracy trade-off: The control participant with the fastest reaction times made the most errors and the control participant with the slowest reaction times made the fewest errors.

2 Note that even with a less-conservative α level of .05, there were only two values that reached significance. One participant (C1) showed a main effect for distractor, *F*(1, 438) = 3.952, *p* = .047, partial η^2 ^= .009, with a trend toward faster responses when the distractor was on an incongruent colour compared with a congruent colour. One participant (C10) showed an interaction between target and distractor, *F*(1, 434) = 4.575, *p* = .033, partial η^2 ^= .01). For this participant, the trend was as follows: with a congruent target, reaction times were faster with an incongruent (than congruent) distractor, whereas with an incongruent target, reaction times were faster with a congruent (than incongruent) distractor. For both of these participants, the effect size was small.

## References

[bibr1-2041669517736323] Banissy M. J. Cohen Kadosh R. C. Maus G. W. Walsh V. Ward J. (2009) Prevalence, characteristics and a neurocognitive model of mirror-touch synaesthesia. Experimental Brain Research 198: 261–272. 19412699 10.1007/s00221-009-1810-9

[bibr2-2041669517736323] Beeli G. Esslen M. Jäncke L. (2005) Synaesthesia: When coloured sounds taste sweet. Nature 434: 38–38. 15744291 10.1038/434038a

[bibr3-2041669517736323] Brainard D. H. (1997) The psychophysics toolbox. Spatial Vision 10: 433–436. 9176952

[bibr4-2041669517736323] Carriere J. Malcolmson K. Eller M. Kwan D. Reynolds M. Smilek D. (2007) Personifying inanimate objects in Synaesthesia. Journal of Vision 7: 532. 10.1162/jocn.2007.19.6.981 17536968

[bibr5-2041669517736323] Flournoy T. (1893) Des phénomènes de synopsie* [Of Synoptic Phenomena]*, Felix Alcan: Paris.

[bibr6-2041669517736323] Glasser, S. (2015). The impact of idiopathic synaesthesia on musical abilities. In R. Timmers, N. Dibben, Z. Eitan, R. Granot, T. Metcalfe, A. Schiavio, & V. Williamson (Eds.), *Proceedings of ICMEM 2015: International conference on the multimodal experience of music*. Sheffield, England: HRI Online Publications, 2015. Retrieved from https://www.hrionline.ac.uk/openbook/chapter/ICMEM2015-Glasser.

[bibr7-2041669517736323] Hubbard E. M. Ramachandran V. S. (2005) Neurocognitive mechanisms of synesthesia. Neuron 48: 509–520. 16269367 10.1016/j.neuron.2005.10.012

[bibr8-2041669517736323] Langan J. Peltier S. J. Bo J. Fling B. W. Welsh R. C. Seidler R. D. (2010) Functional implications of age differences in motor system connectivity. Frontiers in Systems Neuroscience 4: 17. 20589101 10.3389/fnsys.2010.00017 PMC2893009

[bibr9-2041669517736323] Lupiánez J. Callejas A. (2006) Automatic perception and synaesthesia: Evidence from colour and photism naming in a Stroop-negative priming task. Cortex 42: 204–212. 16683494 10.1016/s0010-9452(08)70345-9

[bibr10-2041669517736323] Mattingley J. B. Rich A. N. Yelland G. Bradshaw J. L. (2001) Unconscious priming eliminates automatic binding of colour and alphanumeric form in synaesthesia. Nature 410: 580–582. 11279495 10.1038/35069062

[bibr11-2041669517736323] Meier B. Rothen N. (2013) Grapheme-colour synaesthesia is associated with a distinct cognitive style. Frontiers in Psychology 4: 76–82. 24065938 10.3389/fpsyg.2013.00632 PMC3777024

[bibr12-2041669517736323] Mills C. B. (1999) Digit synaesthesia: A case study using a Stroop-type test. Cognitive Neuropsychology 16: 181–191.

[bibr13-2041669517736323] Nikolić D. Jürgens U. M. Rothen N. Meier B. Mroczko A. (2011) Swimming-style synesthesia. Cortex 47: 874–879. 21402377 10.1016/j.cortex.2011.02.008

[bibr14-2041669517736323] Orne M. T. (1959) The nature of hypnosis: Artefact and essence. Journal of Abnormal Social Psychology 58: 277–299. 10.1037/h0046128 13653876

[bibr15-2041669517736323] Orne M. T. (1962) On the social psychology of the psychological experiment: With particular reference to demand characteristics and their implications. American Psychologist 17: 776–783.

[bibr116-2041669517736323] Pautzke, R. (2010). ‘Making sense” messing around with black boxes: synaesthesia and learning: how to master the Theremin without notes. *Paper presented at the 2010 Meeting of the UK Synaesthesia Association*, Brighton, UK.

[bibr16-2041669517736323] Radvansky G. A. Gibson B. S. McNerney M. (2011) Synesthesia and memory: Color congruency, von Restorff, and false memory effects. Journal of Experimental Psychology: Learning, Memory, and Cognition 37: 219. 10.1037/a0021329 21244115

[bibr17-2041669517736323] Rothen N. Nikolić D. Jürgens U. M. Mroczko-Wąsowicz A. Cock J. Meier B. (2013) Psychophysiological evidence for the genuineness of swimming-style colour synaesthesia. Consciousness and Cognition 22: 35–46. 23247309 10.1016/j.concog.2012.11.005

[bibr18-2041669517736323] Rothen N. Meier B. Ward J. (2012) Enhanced memory ability: Insights from synaesthesia. Neuroscience & Biobehavioral Reviews 36: 1952–1963. 22634573 10.1016/j.neubiorev.2012.05.004

[bibr19-2041669517736323] Rothen N. Meier B. (2010) Grapheme–colour synaesthesia yields an ordinary rather than extraordinary memory advantage: Evidence from a group study. Memory 18: 258–264. 20169501 10.1080/09658210903527308

[bibr20-2041669517736323] Simner J. (2012) Defining synaesthesia. British Journal of Psychology 103: 1–15. 22229768 10.1348/000712610X528305

[bibr21-2041669517736323] Simner J. Holenstein E. (2007) Ordinal linguistic personification as a variant of synaesthesia. Journal of Cognitive Neuroscience 19: 694–703. 17381259 10.1162/jocn.2007.19.4.694

[bibr22-2041669517736323] Simner J. Mayo N. Spiller M. J. (2009) A foundation for savantism? Visuo-spatial synaesthetes present with cognitive benefits. Cortex 45: 1246–1260. 19665699 10.1016/j.cortex.2009.07.007

[bibr23-2041669517736323] Smilek D. Malcolmson K. A. Carriere J. S. Eller M. Kwan D. Reynolds M. (2007) When “3” is a jerk and “E” is a king: Personifying inanimate objects in synesthesia. Journal of Cognitive Neuroscience 19: 981–992. 17536968 10.1162/jocn.2007.19.6.981

[bibr124-2041669517736323] Stroop, J. R. (1935). Studies of interference in serial verbal reactions. *Journal of Experimental Psychology*, *18*, 643–662.

[bibr24-2041669517736323] Ward J. Huckstep B. Tsakanikos E. (2006) Sound-colour synaesthesia: To what extent does it use cross-modal mechanisms common to us all? Cortex 42: 264–280. 16683501 10.1016/s0010-9452(08)70352-6

[bibr25-2041669517736323] Ward J. Simner J. (2003) Lexical-gustatory synaesthesia: Linguistic and conceptual factors. Cognition 89: 237–261. 12963263 10.1016/s0010-0277(03)00122-7

[bibr26-2041669517736323] Watson M. R. Akins K. A. Spiker C. Crawford L. Enns J. T. (2014) Synesthesia and learning: A critical review and novel theory. Frontiers in Human Neuroscience 8: 98. 24592232 10.3389/fnhum.2014.00098 PMC3938117

[bibr27-2041669517736323] Watson M. R. Chromy J. Crawford L. Eagleman D. M. Enns J. T. Akins K. A. (2017) The prevalence of synaesthesia depends on early language learning. Consciousness and Cognition 48: 212–231. 28013176 10.1016/j.concog.2016.12.004

[bibr28-2041669517736323] White R. C. Plassart A. (2015) Stimulus-parity synaesthesia (1893/2014): Introducing a ‘forgotten’ subtype. Cortex 66: 146–148. 25603920 10.1016/j.cortex.2014.09.023

[bibr29-2041669517736323] White, R. C., Dumbalska, M., Duta, M. M., & Nation, K. (in press). ‘17’ is odd and ‘seventeen’ is even: Meaning and physical form in stimulus-parity synaesthesia. *Quarterly Journal of Experimental Psychology*. 10.1177/1747021817738712 30117381

[bibr30-2041669517736323] Wollen K. A. Ruggiero F. T. (1983) Colored letter synesthesia. Journal of Mental Imagery 7: 83–86.

[bibr31-2041669517736323] Woods D. L. Wyma J. M. Yund E. W. Herron T. J. Reed B. (2015) Factors influencing the latency of simple reaction time. Frontiers in Human Neuroscience 9: 131. 25859198 10.3389/fnhum.2015.00131 PMC4374455

[bibr32-2041669517736323] Yaro C. Ward J. (2007) Searching for Shereshevskii: What is superior about the memory of synaesthetes? The Quarterly Journal of Experimental Psychology 60: 681–695. 17455076 10.1080/17470210600785208

